# Assessment of the practices of prevention and bloodstream infection control associated to a central venous catheter of short permanence where of clinical indicators

**DOI:** 10.1186/1753-6561-5-S6-P55

**Published:** 2011-06-29

**Authors:** JM Jardim, RA Lacerda

**Affiliations:** 1 Surgery-Medical, Escola de Enfermagem da USP - EEUSP, São Paulo, Brazil

## Introduction / objectives

The aim of this study was evaluate the conformity of the prevention practices and the bloodstream infection control associated to central venous catheter of short permanence.

## Methods

Control, prospective and observational study, with the aim to evaluate the program, practices and procedures performance related to an insertion and maintenance of central venous catheter. This study was realized in the surgical intensive care unit and in surgical center of to a Public Teaching Hospital, in São Paulo, Brazil and was between August and November of 2010. The data collection used direct observation and analysis of registration records. The professionals involved were doctors and nurses.

## Results

Were realized 4719 observations. The results show: a) absence of conformity (0,0%) during the evaluation of the adherence to specific measures of prevention and control of bloodstream infection associated to a central venous catheter, the most responsible for the not using the occlusive dressing at the end of the insertion of central venous catheter; b) 45,6% of conformity in hubs disinfection and connectors before the manipulation with alcoholic chlorhexidine; c) 110% of agreement in change of equips and transducers with the manufactures instructions; d) 8,1% of hit when were evaluated the opportunities of hand hygiene before and after the manipulation venous central catheter.

## Conclusion

New educational and training programs need to be realized to improve the practices and bloodstream infection control associated to venous central catheter in this surgical intensive care unit.

## Disclosure of interest

None declared.

